# Copper Nanoparticles at Optimized Concentrations Exhibit Minimal Intrinsic Biological Effects in Hepatocellular Carcinoma Cells

**DOI:** 10.3390/biomedicines14102212

**Published:** 2026-09-30

**Authors:** Erkan Kahraman, Tarık İnci, Hakan Turan Kiriş, Yeliz Yıldırım, Erdem Göker, Burcu Cakar

**Affiliations:** 1 Atatürk Vocational School of Health Services, Ege University, Izmir 35100, Türkiye; erkan.kahraman@ege.edu.tr; 2 Department of Internal Medicine, Faculty of Medicine, Ege University, Izmir 35100, Türkiye; trkinci15@gmail.com (T.İ.); hakantkiris@gmail.com (H.T.K.); 3 Department of Chemistry, Faculty of Science, Ege University, Izmir 35040, Türkiye; yeliz.yildirim@ege.edu.tr; 4 Faculty of Medicine, Medical Oncology, Ege University, Izmir 35100, Türkiye; erdem.goker@ege.edu.tr

**Keywords:** copper, copper nanoparticles, cancer, hepatocellular carcinoma, nano drug, nanomedicine

## Abstract

**Background**: Hepatocellular carcinoma (HCC) is the most common primary liver malignancy and remains a major cause of cancer-related morbidity and mortality worldwide; recent advancements in nanomedicine provide promising prospects for its treatment. Studies in the field of nanomedicine in recent years emphasize that copper nanoparticles (CuNPs) can be used as significant drug carriers in liver cancer. However, the excessive variability in their cytotoxic profiles restricts the safe use of these particles as drug delivery vehicles. Our study aimed to determine concentration ranges of CuNPs with minimal detectable effects on cellular viability and homeostasis in a liver cancer cell model. **Methods**: The cytotoxic potential of ~50 nm CuNPs synthesized via a chemical reduction method (NaBH_4_/PVP) was evaluated in well-differentiated Huh7 and poorly differentiated Mahlavu cell lines. **Results**: SEM analysis revealed CuNPs with particle sizes of approximately 42–55 nm (~50 nm), while DLS measurements showed hydrodynamic diameters of 60–80 nm. Cell viability assays demonstrated that CuNPs reduced cell viability in Huh7 and Mahlavu cells in a dose- and time-dependent manner. Optimized concentrations at which cell viability was not significantly affected were defined as 0.04 µg/mL for Huh7 and 0.1 µg/mL for Mahlavu. At these specific doses, colony-forming capacity was not significantly affected, apoptosis was not induced in HCC cells, and cellular morphology remained preserved. Furthermore, pAKT(Ser473)/AKT signaling was not significantly altered in Mahlavu cells at the optimized concentration, whereas pAKT(Ser473) was not reliably detectable in Huh7 cells under the experimental conditions. The levels of the pro-apoptotic protein cleaved caspase-3 were not significantly altered at the optimized concentrations. **Conclusions**: Our findings indicate that, at optimized concentrations, CuNPs exhibit minimal intrinsic biological effects on HCC cells, supporting their further investigation as potential drug-delivery platforms for HCC.

## 1. Introduction

Cancer remains a major global health burden and one of the leading causes of mortality worldwide [1,2]. Despite substantial advances in cancer treatment, therapeutic efficacy remains limited in many advanced and metastatic malignancies, while systemic toxicity, off-target effects, and the development of drug resistance continue to pose major challenges to effective treatment [3,4,5]. Among these malignancies, hepatocellular carcinoma (HCC) represents a particularly challenging disease due to its high mortality, frequent presentation at advanced stages, and limited therapeutic options. Nanotechnology-based approaches developed to overcome these limitations represent a paradigm shift aimed at enhancing bioavailability and minimizing side effects by delivering therapeutic agents directly to tumor tissue [6]. Today, a wide variety of nanoparticles, each designed for specific therapeutic targets, are utilized in cancer research. Among these, organic structures such as liposomes, polymeric nanoparticles, micelles, and dendrimers stand out for their biocompatibility, whereas inorganic structures such as silica, carbon nanotubes, graphene, and quantum dots offer high stability and durability [7]. In particular, metallic nanoparticles are utilized in cancer therapy, drug delivery, and tumor imaging applications due to their large surface areas and flexibility in size and morphology [8].

Copper nanoparticles (CuNPs) have emerged as promising metallic nanomaterials for cancer diagnosis and therapy due to their relatively low production cost, broad availability, and favorable catalytic properties compared with other metallic nanoparticles, such as gold and silver [9]. As copper is an essential trace element involved in numerous physiological and intracellular processes, CuNPs have attracted increasing interest for biomedical applications [10,11]. In oncology, CuNPs may exert anticancer effects through multiple mechanisms, including the generation of reactive oxygen species within the tumor microenvironment and the induction of copper-dependent cell death pathways. Of particular interest is cuproptosis, a recently characterized form of regulated cell death driven by the accumulation of intracellular copper and its interaction with lipoylated proteins of the mitochondrial tricarboxylic acid cycle [12,13]. Furthermore, the high demand for copper in cancer cells due to their increased metabolic rates allows CuNP-based therapies to target cancer cells more selectively than normal cells [14]. However, the cytotoxic effects of CuNPs, ranging widely from microgram to milligram levels, have been reported depending on the synthesis method and particle size [9]. Therefore, the therapeutic dose levels of CuNPs cannot be fully standardized due to major variations in their nanotoxicological profiles [15,16]. This situation highlights the need to define specific concentration ranges in which CuNPs exert minimal intrinsic effects on cellular homeostasis on their own, thereby enabling CuNPs to be used as vehicles for drug delivery systems.

The liver is of particular relevance in nanomedicine, as it represents a major site of nanoparticle accumulation following systemic administration, with reported hepatic uptake varying considerably depending on nanoparticle characteristics [17]. In this context, CuNPs have attracted increasing interest for potential applications in hepatocellular carcinoma. Previous studies have demonstrated that CuNPs can exert dose-dependent cytotoxic and antiproliferative effects on HCC cells. In addition to their intrinsic biological activity, CuNPs can be functionalized with targeting ligands or therapeutic agents, offering the potential for selective drug delivery and improved therapeutic efficacy [9]. The tendency of nanoparticles to be sequestered and accumulated by cells of the hepatic reticuloendothelial system makes the liver both an attractive target for nanoparticle-based therapies and a critical organ for evaluating potential nanoparticle-associated toxicity [18]. In particular, the high affinity of metallic structures such as copper nanoparticles for liver tissue and their capacity to trigger oxidative stress in a dose-dependent manner make it essential to define the biocompatibility limits of these particles in liver cancer models [19]. However, the effects of low-dose CuNPs on cellular viability and homeostasis in HCC cells remain insufficiently characterized, highlighting the need to identify concentrations with minimal intrinsic biological effects. Thus, defining such concentrations is an important step toward their potential use in drug-delivery systems. In contrast to previous studies in HCC models that have primarily focused on the cytotoxic and anticancer effects of CuNPs at higher concentrations, the present study specifically aimed to define low-dose concentrations at which CuNPs exert minimal detectable effects on HCC cells [20,21].

## 2. Materials and Methods

### 2.1. Reagents

Copper(II) sulfate pentahydrate (CuSO_4_·5H_2_O, Merck, Darmstadt, Germany), sodium borohydride (NaBH_4_, Merck), polyvinylpyrrolidone (PVP, MW ≈ 10,000, Sigma-Aldrich, St. Louis, MO, USA), and sodium carbonate (Na_2_CO_3_, Merck) were used without further purification. All solutions were prepared using deionized water.

### 2.2. Synthesis of Copper Nanoparticles

CuNPs were synthesized via a chemical reduction method using sodium borohydride (NaBH_4_) as the reducing agent and polyvinylpyrrolidone (PVP) as the stabilizing agent in aqueous media, following modified procedures reported by Aguilar et al. [22]. Briefly, 50 mL of 0.1 M CuSO_4_·5H_2_O solution was mixed with 0.2 g of PVP under continuous magnetic stirring. Subsequently, 10 mL of 0.01 M sodium carbonate solution was added, followed by the dropwise addition of 10 mL of freshly prepared 0.1 M NaBH_4_ solution at room temperature. The reaction mixture immediately changed from light blue to orange-brown, indicating the formation of CuNPs [23]. The reaction was maintained under continuous stirring for 30 min at room temperature. The resulting colloidal suspension was centrifuged at 10,000 rpm for 10 min. The collected precipitate was washed successively with deionized water, ethanol, and acetone to remove residual reagents and then vacuum-dried at 60 °C for 12 h. The morphology of the synthesized CuNPs was examined by scanning electron microscopy (SEM). Prior to imaging, the samples were mounted on carbon adhesive tape, sputter-coated with a thin layer of gold, and analyzed using a Zeiss Gemini 500 scanning electron microscope (Carl Zeiss Microscopy GmbH, Jena, Germany). The hydrodynamic particle size of the synthesized CuNPs was determined by dynamic light scattering (DLS) at 25 °C using a Malvern Zetasizer Ultra (Malvern Panalytical Ltd., Malvern, UK) with a 173° backscattering detection angle. All measurements were performed in triplicate.

### 2.3. Cell Culture

The well-differentiated Huh7 and poorly differentiated Mahlavu human hepatocellular carcinoma cell lines were used to evaluate the cellular effects of CuNPs. These cell lines were obtained as a kind gift from Dr. Neşe Atabey. The HCC cells were cultured in Dulbecco’s Modified Eagle Medium (DMEM, Biowest, Nuaillé, France, Cat. No: B.L0064) supplemented with 10% FBS (Fetal Bovine Serum, Biowest, Cat. No: B.S160H), 2 mM L-glutamine (Biowest, Cat. No: B.X0550), 100 U/mL penicillin, 0.1 mg/mL streptomycin (Biowest, Cat. No: B.L0022), and 1% MEM non-essential amino acids solution (Biowest, Cat. No: B.X0557), and incubated at 37 °C in a humidified atmosphere with 5% CO_2_.

### 2.4. CuNP Treatment

To prepare the nanoparticle solution, copper nanoparticles were suspended in DMSO (Dimethyl sulfoxide) at a concentration of 10 mg/mL, followed by vigorous pipetting for approximately 5 min. The suspension was then sonicated to ensure full dispersion and minimize particle aggregation. An intermediate CuNP stock solution at 1 mg/mL was subsequently prepared by diluting the 10 mg/mL stock in complete culture medium. This intermediate stock was further diluted in complete culture medium to obtain the predetermined experimental concentrations. DMSO was added to the corresponding control groups as a vehicle control at concentrations equivalent to those present in the CuNP-treated groups. For Huh7 cells treated with 0.04 µg/mL CuNPs and Mahlavu cells treated with 0.1 µg/mL CuNPs, the final DMSO concentrations were 0.0004% and 0.001%, respectively. For the highest CuNP concentration used in the IC50 experiment (10 µg/mL), the corresponding final DMSO concentration was 0.1%.

### 2.5. Cell Viability Assay

Cell viability was evaluated using the MTT (3-(4,5-dimethylthiazol-2-yl)-2,5-diphenyl-2H-tetrazolium bromide) assay. Briefly, HCC cells were seeded into 96-well plates at a density of 2500 cells per well. Following a 24 h incubation period, the culture medium was replaced with fresh medium containing the prepared copper nanoparticle solution at concentrations ranging from 0 to 10 µg/mL and the plates were incubated for an additional 24 h. A cell-free control containing CuNPs was also included to check whether CuNPs interfered with the MTT assay. At the end of the incubation period, a 15 μL MTT (Sigma-Aldrich, Cat. No: M5655) solution (5 mg/mL in PBS) was added to each well, and the cells were incubated for 4 h. The culture medium was then discarded, and 100 µL of DMSO was added to each well to dissolve the formazan crystals. The absorbance was measured at a wavelength of 570 nm using a microplate reader (DTX 880 Multimode Detector, Beckman Coulter Inc., Brea, CA, USA). Cell viability was calculated using the following formula:

Cell viability (%) = (OD of treated sample-blank : OD of untreated sample-blank) × 100

Dose–response analysis was performed using GraphPad Prism 8.0 software to calculate the half-maximal inhibitory concentration (IC50) values for each treatment group. To determine the non-toxic threshold of the nanoparticles, a concentration range of 0 to 0.5 µg/mL was selected. The HCC cells were subsequently treated with these doses for 24, 72, and 120 h, and an MTT assay was performed to evaluate both time- and dose-dependent cell viability. The highest nanoparticle dose that did not statistically affect cell viability was determined.

### 2.6. Colony Formation Assay

Hepatocellular carcinoma cells were seeded into 35 mm tissue culture dishes at a concentration of 100 cells/dish. Complete media containing CuNPs were treated to the cells for 120 h at concentrations of 0.04 µg/mL for HuH-7 and 0.1 µg/mL for Mahlavu cells. After 120 h, the CuNP-treated media were replaced with fresh media. The cultures were incubated for 10 days, and the culture media were renewed with fresh media every two days. At the end of the incubation period, colony formations were observed in the culture plates. Crystal violet staining was performed to visualize the colonies. Briefly, the cell culture mediums were discarded, and the cells were washed twice with PBS (Phosphate-Buffered Saline, Biowest, Cat. No: B.L0615). The colonies were fixed in methanol and stained with crystal violet solution (Sigma, Cat. No: V5265) for 10 min. Finally, the plates were washed three times with distilled water, and the colony number in each plate was counted.

### 2.7. Apoptosis Analysis

To identify apoptotic cells, acridine orange (Sigma, Cat. No. 235474) and ethidium bromide (Sigma, Cat. No. E7634) dual staining was utilized. In brief, HCC cells were seeded into 96-well culture plates at a density of 2500 cells/well. After the cells adhered, they were incubated with CuNPs for a 120 h period. At the end of the treatment, a combined solution of acridine orange (100 µg/mL) and ethidium bromide (100 µg/mL) was introduced into each well. Finally, the cells were observed using a fluorescence microscope (Leica DM IL, Leica Microsystems CMS GmbH, Wetzlar, Germany) to evaluate apoptotic alterations, according to Ribble et al. [24].

### 2.8. Giemsa Staining

Giemsa staining was performed to investigate cellular morphological alterations. Following the 120 h CuNP treatment, the culture medium was discarded, and the cells were rinsed three times with PBS. Cell fixation was carried out using 100% methanol for 10 min. After removing the methanol, the cells were incubated with Giemsa stain (Sigma, Cat. No. GS500) for an additional 10 min. Following the staining process, residual dye was removed by washing the cells three times with distilled water. Morphological variations in the cells were analyzed using bright-field microscopy.

### 2.9. Western Blot Analysis

Total protein was isolated from HCC cells utilizing a radioimmunoprecipitation assay (RIPA) buffer containing 150 mM NaCl, 50 mM Tris-Cl (pH 7.4), 1 mM EDTA (pH 8.0), 1% NP-40, 1X protease inhibitor cocktail, 1 mM NaF, and 1 mM Na_3_VO_4_. Protein quantification was carried out using the Pierce BCA protein assay kit (Thermo Fisher Scientific, Rockford, IL, USA, Cat. No. 23225) in accordance with the manufacturer’s protocol. Absorbance was determined at 562 nm via a microplate reader (DTX 880 Multimode Detector, Beckman Coulter Inc.). Equal amounts of protein lysates were separated on SDS-polyacrylamide gels and subsequently transferred onto PVDF membranes. To prevent non-specific binding, membranes were blocked for 1 h at room temperature with a 5% blocking solution (5% BSA in TBS-T). The membranes were then incubated overnight at 4 °C with primary antibodies against phospho-AKT (Ser473) (Cell Signaling, Danvers, MA, USA, Cat. No. CS-4060), AKT (Cell Signaling, Cat. No. CS-2920), Caspase 3 (Cell Signaling, Cat. No. 9662) and Calnexin (Santa Cruz, Cat. No. sc-23954) at a 1:1000 dilution in 3% BSA. Following primary antibody incubation, the membranes were treated with appropriate secondary antibodies (Thermo Fisher Scientific, Cat. No. 23430 and Cat. No. 23460) for 1 h at room temperature. Protein bands were visualized using the enhanced chemiluminescence (ECL) detection method and captured with a Kodak Gel Logic 2200 Imaging System. The densitometric analysis of the bands was performed using ImageJ 1.54g software.

### 2.10. Statistical Analysis

All statistical analyses were conducted using GraphPad Prism 8.4 software. For comparisons involving more than two groups, one-way analysis of variance (ANOVA) followed by Dunnett’s post hoc test was performed. Student’s *t*-test was used to determine the significance of differences between two groups. All experiments were performed independently at least three times, and the data were expressed as mean ± standard deviation (SD). Statistical significance was defined as *p* < 0.05.

## 3. Results

### 3.1. Morphology and Particle Size Analysis of Synthesized CuNPs

**Figure 1:**
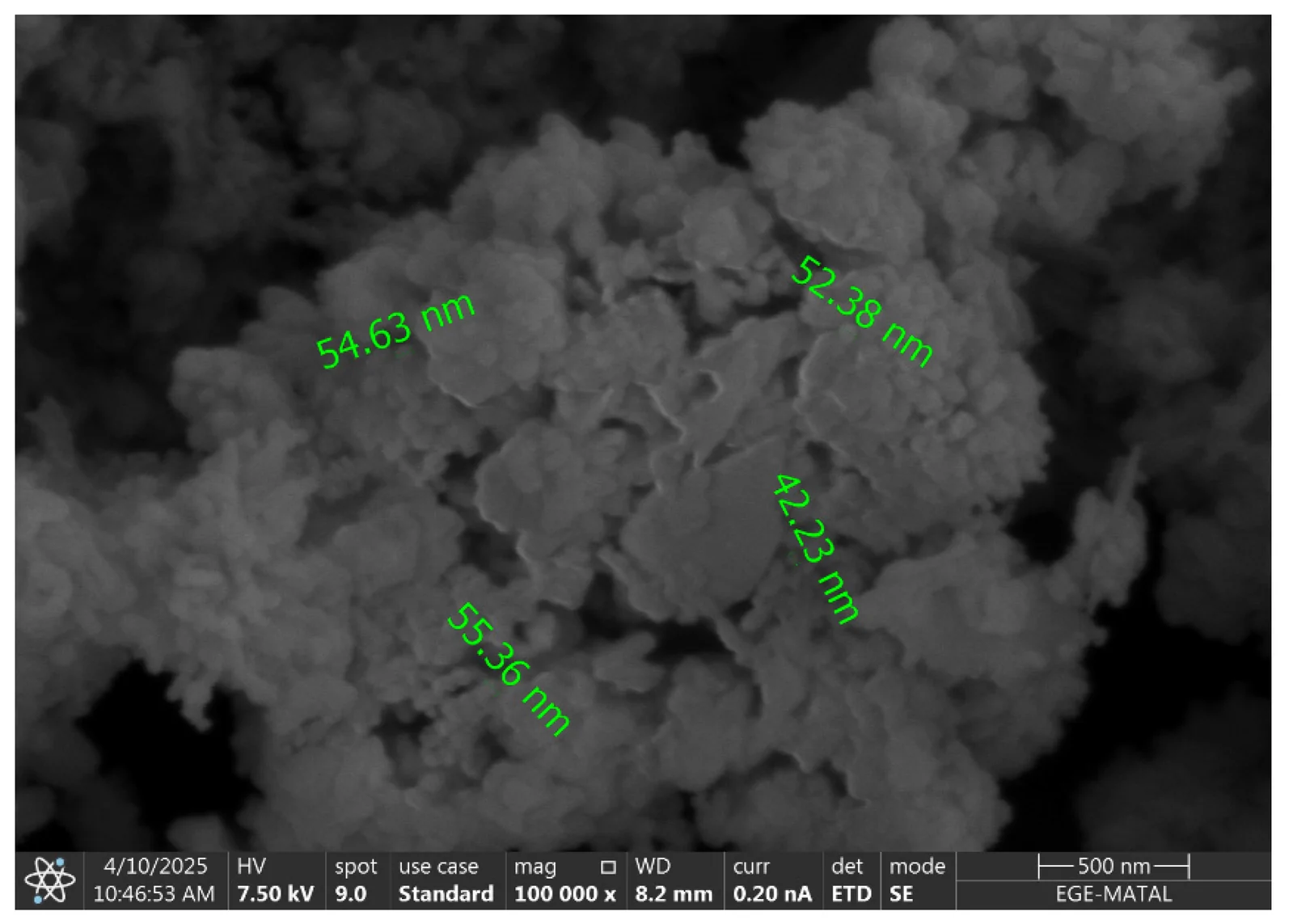
SEM image of the synthesized CuNPs at 100,000× magnification.

**Figure 2:**
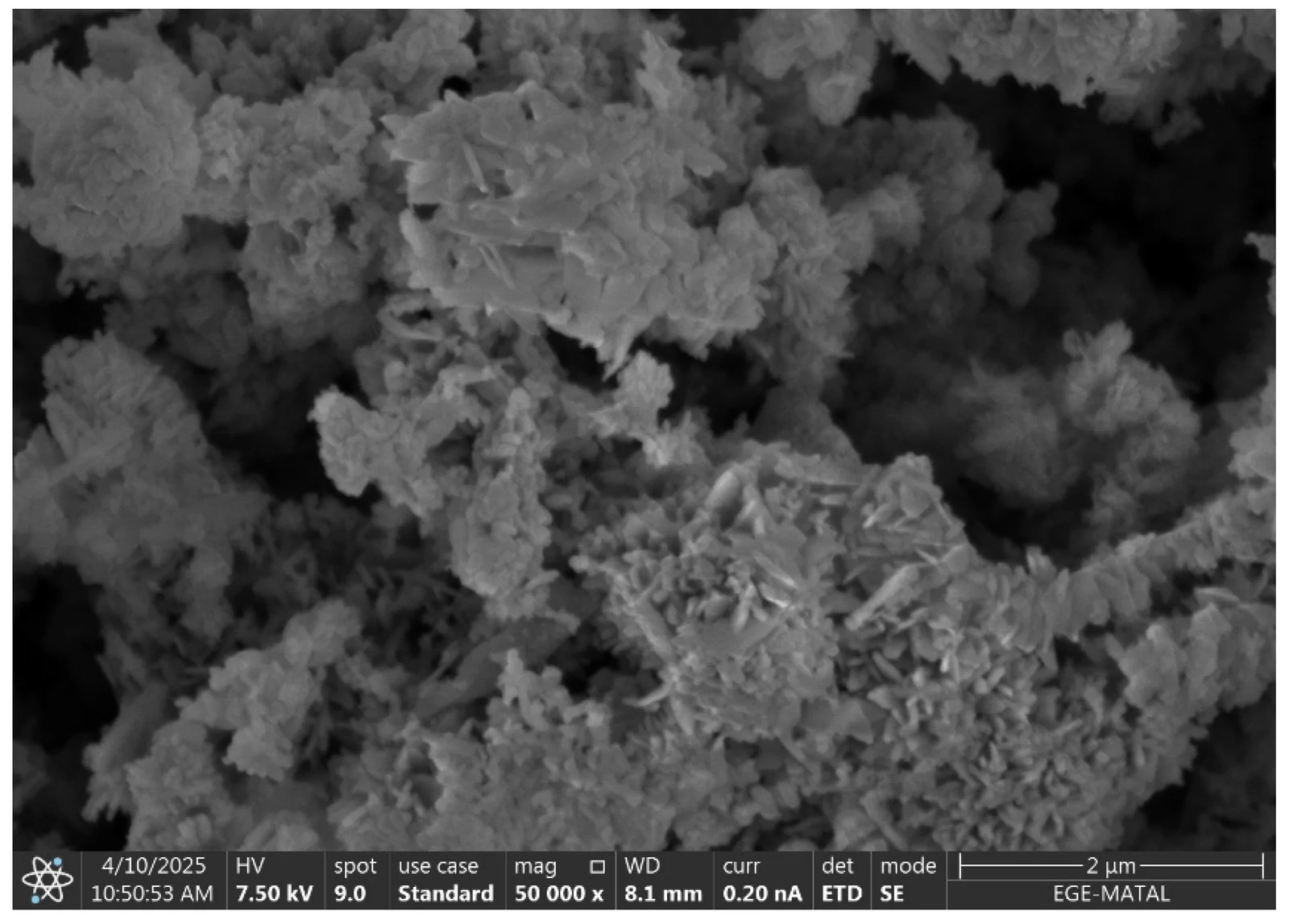
SEM image of the synthesized CuNPs at 50,000× magnification.

The morphology of the synthesized copper nanoparticles (CuNPs) was investigated using scanning electron microscopy (SEM). Representative SEM micrographs of the CuNPs synthesized under the optimized reaction conditions are presented in Figure 1 and Figure 2. Figure 1 presents spherical copper nanoparticles with relatively uniform size distribution and moderate agglomeration. The nanoparticles appear to be relatively well separated, indicating successful nanoparticle formation under the selected synthesis conditions. Figure 2 shows well-defined spherical particles with sharper boundaries and improved dispersion. Compared with Figure 1, the nanoparticles appear more uniformly distributed with reduced clustering.

The SEM images showed particle dimensions of approximately 42–55 nm, corresponding to an approximate particle size of 50 nm. In contrast, DLS measurements yielded hydrodynamic diameters of 60–80 nm. This difference is consistent with the distinct measurement principles of the two techniques: SEM evaluates the physical dimensions of particles in the dried state, whereas DLS determines the hydrodynamic diameter of particles dispersed in a liquid medium. Consequently, DLS values may be slightly higher because they reflect the hydrated/solvated state of the particles and their hydrodynamic behavior in dispersion [25,26]. Similar technique-dependent differences between dry-state particle-size measurements and DLS-derived hydrodynamic diameters have also been reported for CuO nanoparticles [26].

### 3.2. CuNPs Inhibited HCC Cell Survival and Exhibited Concentration-Dependent Cytotoxicity in Huh7 and Mahlavu Cells

**Figure 3:**
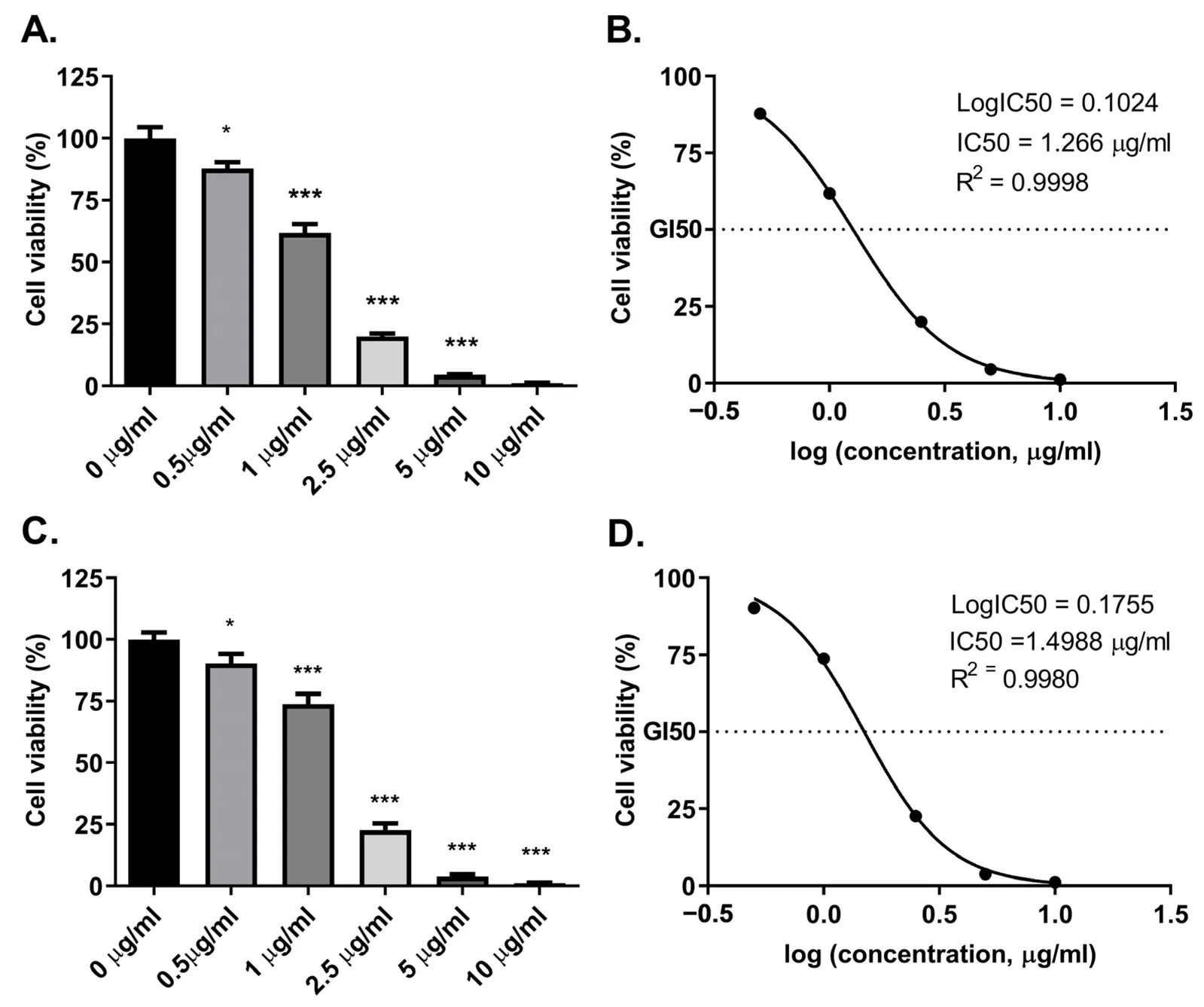
CuNPs inhibit cell survival and exhibit concentration-dependent cytotoxicity in Huh7 and Mahlavu cells. Cell viability of Huh7 (**A**) and Mahlavu (**C**) cells treated with indicated concentrations of CuNPs (0.5, 1, 2.5, 5, and 10 µg/mL) for 24 h was determined by MTT assay. (**B**,**D**) Dose–response curves and the calculated IC50 values for Huh7 (**B**) and Mahlavu (**D**) cell lines. The LogIC50 and R^2^ values are indicated on each plot. Statistical analysis revealed that significant growth inhibition was initiated at 0.5 µg/mL, reaching maximum significance at concentrations above 1 µg/mL in both cell models. Results are representative of three independent experiments and expressed as the means ± SD (* *p* < 0.05, *** *p* < 0.001 compared with untreated control).

To evaluate the cytotoxic potential of synthesized copper nanoparticles (CuNPs), human hepatocellular carcinoma (HCC) cell lines—Huh7 (well-differentiated) and Mahlavu (poorly differentiated)—were treated with varying concentrations of CuNPs (0–10 µg/mL) for 24 h. CuNP treatment resulted in a dose-dependent reduction in cell viability in Huh7 cells (Figure 3A). The IC50 (50% growth inhibition) value for the Huh7 cell line was determined to be 1.266 µg/mL (LogIC50 = 0.1024), with a high correlation coefficient (R^2^ = 0.9998) (Figure 3B). Similarly, the viability of Mahlavu cells was effectively inhibited by CuNP administration in a concentration-dependent manner (Figure 3C). For Mahlavu cells, the IC50 value was calculated as 1.498 µg/mL (LogIC50 = 0.1755; R^2^ = 0.9980) (Figure 3D), suggesting that Mahlavu cells are slightly more resistant to CuNP exposure compared to the Huh7 line. The cell-free CuNP + MTT control showed an absorbance similar to the blank (0.47 ± 0.02 vs. 0.48 ± 0.01, respectively; *p* > 0.05), indicating no detectable interference with the MTT assay. Statistical analysis revealed that while significant growth inhibition was initiated at 0.5 µg/mL (* *p* < 0.05), the cytotoxic effect reached maximum significance at concentrations of 1 µg/mL and above (*** *p* < 0.001) in both HCC cell models.

### 3.3. Time-Course Analysis Identified Optimized Non-Cytotoxic Concentrations in HCC Cells

**Figure 4:**
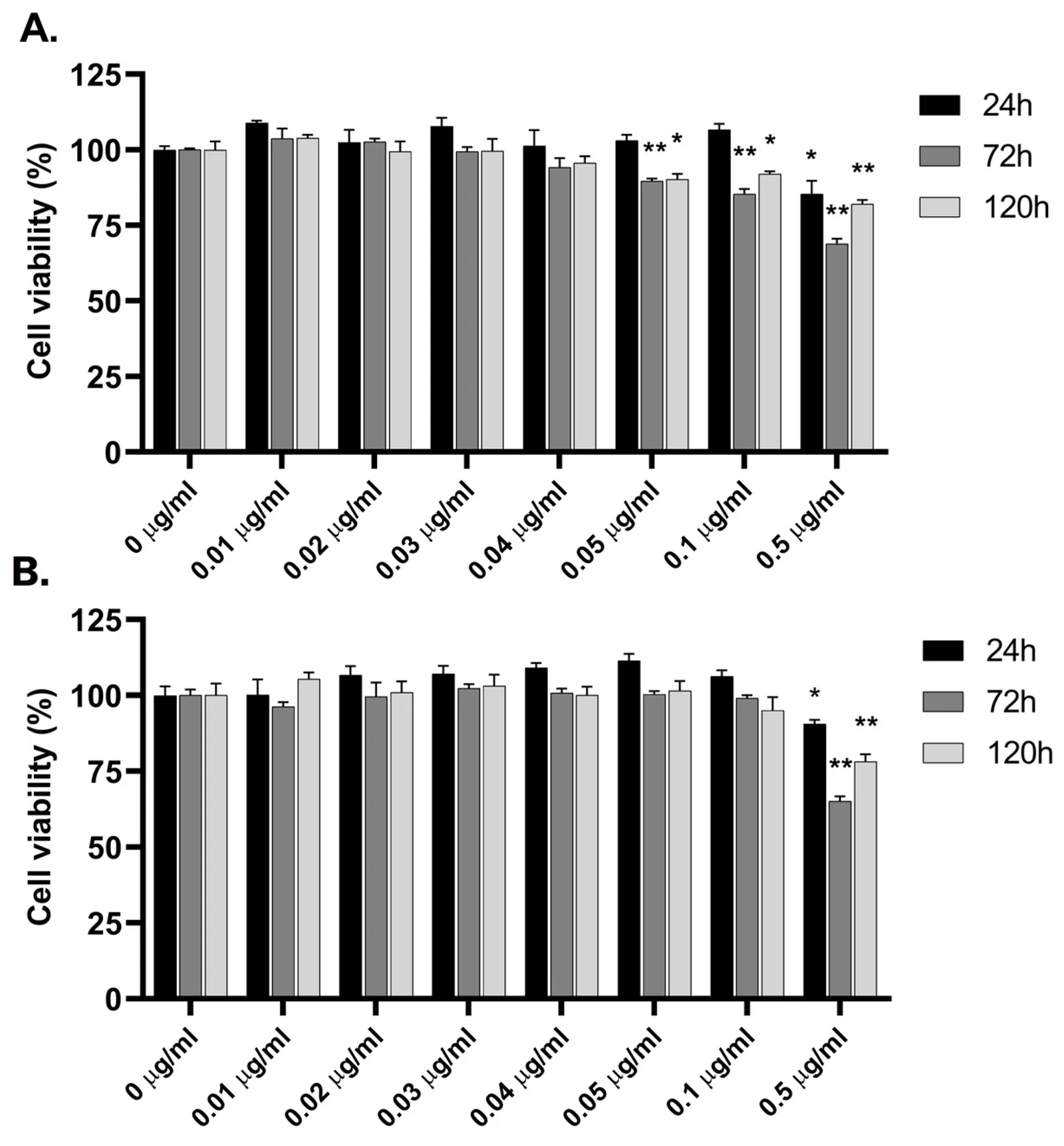
Time-course analysis of optimal non-cytotoxic thresholds for CuNPs in HCC models. Cell viability of Huh7 (**A**) and Mahlavu (**B**) cells following treatment with a range of low-dose CuNPs (0.01, 0.02, 0.03, 0.04, 0.05, 0.1, and 0.5 µg/mL) for 24, 72, and 120 h was assessed by MTT assay. The analysis identified concentrations with minimal detectable effects on cellular viability across the evaluated time points. Significant growth inhibition was observed at 0.05 µg/mL and higher concentrations in Huh7 cells and 0.5 µg/mL in Mahlavu cells, particularly during prolonged exposure (72–120 h). Results are representative of three independent experiments and are expressed as the means ± SD (* *p* < 0.05, ** *p* < 0.01 compared with the untreated control).

To further characterize the effects of CuNP exposure, we evaluated the impact of low-dose CuNP exposure (0.01–0.5 µg/mL) on the viability of well-differentiated Huh7 and poorly differentiated Mahlavu cells at 24, 72, and 120 h (Figure 4). Huh7 cells maintained high viability (>95%) at concentrations up to 0.04 µg/mL across all time points, exhibiting no statistical difference compared to the untreated control group. Significant growth inhibition in Huh7 cells was initiated only at 0.05 µg/mL, after 72 h of exposure (* *p* < 0.05), with cytotoxicity becoming more pronounced at higher concentrations and longer durations (Figure 4A). Therefore, 0.04 µg/mL was identified as the non-cytotoxic threshold for HuH-7 cells.

Similarly, in the poorly differentiated Mahlavu cell line, CuNPs demonstrated a broader biocompatibility window during long-term incubation (Figure 4B). At concentrations ranging from 0.01 to 0.1 µg/mL, Mahlavu cells showed no significant reduction in viability throughout the 24, 72, and 120 h treatment periods. While a significant decrease in cell viability was observed at the highest tested concentration of 0.5 µg/mL (*p* < 0.05), concentrations of 0.1 µg/mL and below did not significantly affect cell viability (*p* > 0.05). Therefore, 0.1 µg/mL was identified as the non-cytotoxic threshold for Mahlavu cells.

### 3.4. Optimized Non-Cytotoxic Concentrations of CuNPs Do Not Impair the Colony Forming Capacity of HCC Cells

**Figure 5:**
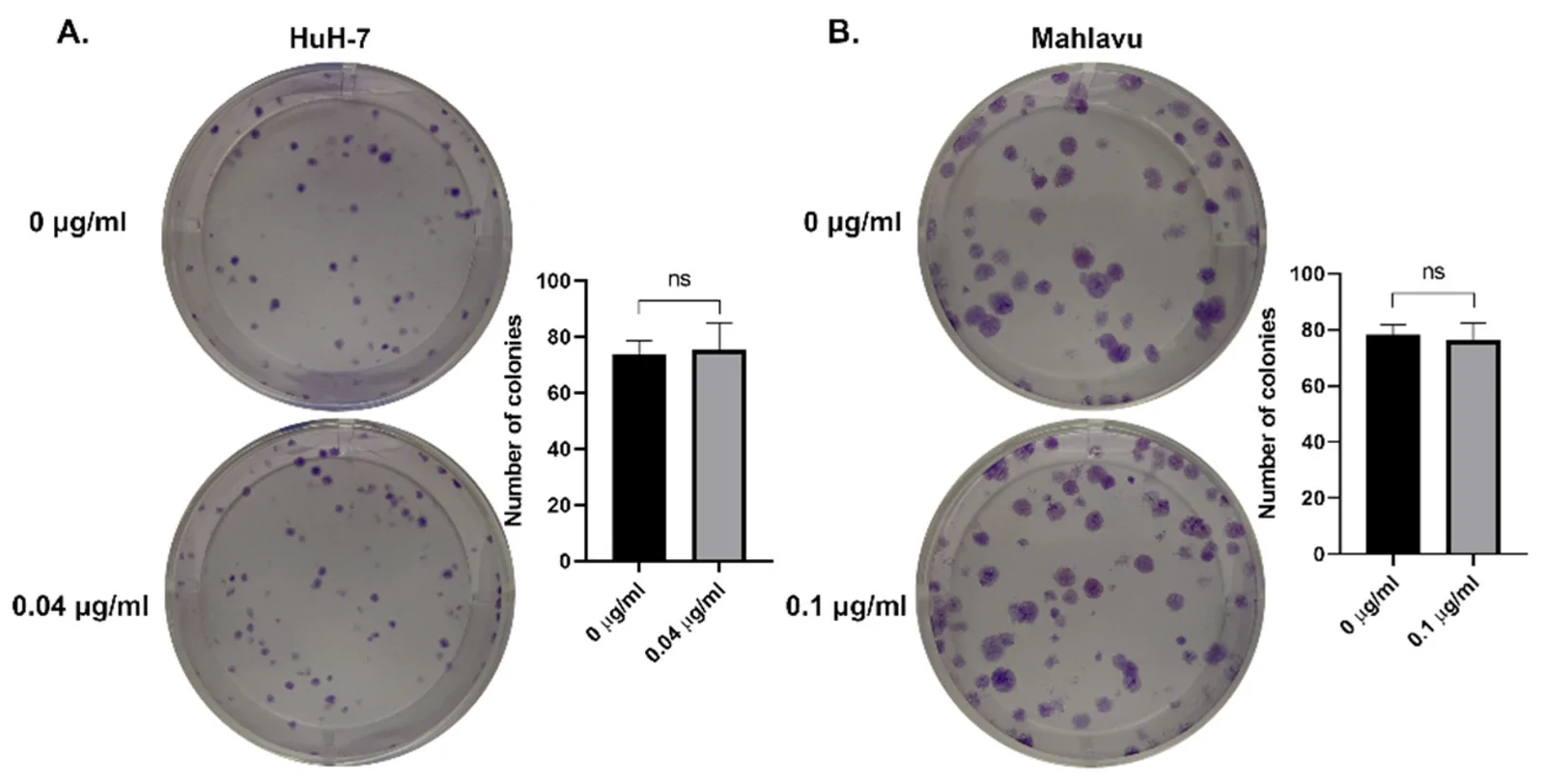
Optimized CuNP concentrations do not interfere with the colony formation ability of Huh7 and Mahlavu cells. Representative images and quantitative analysis of the colony formation assay in Huh7 (**A**) and Mahlavu (**B**) cells. Huh7 cells were treated with 0.04 µg/mL CuNPs, and Mahlavu cells were treated with 0.1 µg/mL CuNPs. Bar graphs represent the average number of colonies counted in each group. The results show no significant difference between the control and the CuNP-treated groups. Data are expressed as the means ± SD from three independent experiments (*p* > 0.05, ns: not significant compared with the untreated control).

To further characterize the effects of the selected concentrations and assess their potential impact on long-term proliferative capacity of HCC cells, we performed a colony formation assay. Huh7 and Mahlavu cells were treated with CuNPs, their respective optimized non-cytotoxic concentrations (0.04 µg/mL for Huh7 and 0.1 µg/mL for Mahlavu). The treatment of Huh7 cells with 0.04 µg/mL CuNPs did not result in a significant reduction in the number of colonies compared to the untreated control group (74.8 ± 10.3 vs. 73.2 ± 5.6, respectively, *p* > 0.05) (Figure 5A). Similarly, the colony-forming ability of the Mahlavu cell line remained statistically non-significant following exposure to 0.1 µg/mL CuNPs (Figure 5B). Quantitative analysis revealed that the number of colonies in the treated Mahlavu group was similar to the control (76.1 ± 6.2 vs. 78.3 ± 3.4, respectively, *p* > 0.05). Collectively, these results demonstrate that at optimized concentrations, CuNPs do not exert inhibitory effects on the colony formation ability of HCC cells.

### 3.5. Optimized Non-Cytotoxic Concentrations of CuNPs Do Not Trigger Apoptosis or Impair Survival Signaling in HCC Cells

**Figure 6:**
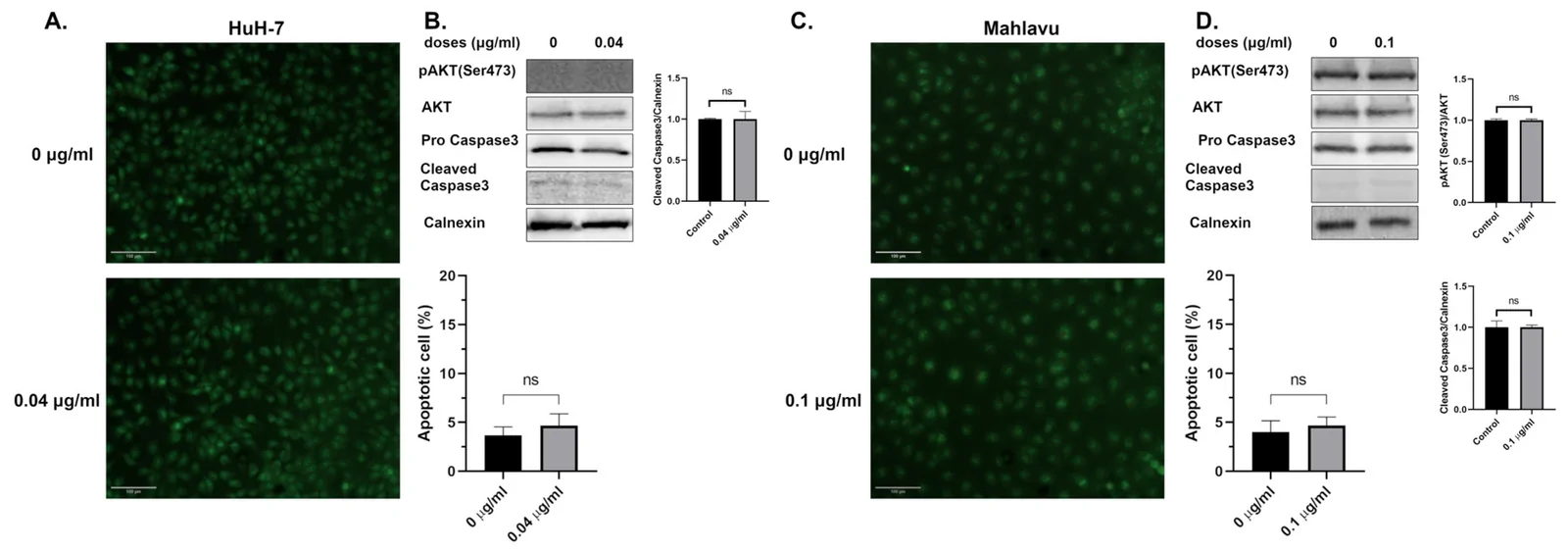
Optimized CuNP concentrations do not induce apoptosis or activate the caspase cascade in HCC cell lines. Representative images and quantitative analysis of AO/EB dual staining in Huh7 (**A**) and Mahlavu (**C**) cells. Cells were treated with their respective optimized doses (0.04 µg/mL and 0.1 µg/mL) for 120 h. Viable cells appear green with intact nuclei. The bar graphs represent the percentage of apoptotic cells, showing no significant difference compared to the control group. Western blot analysis of apoptosis and survival-related proteins in Huh7 (**B**) and Mahlavu (**D**) cells. Protein levels of pAKT(Ser473), AKT, Pro Caspase3, and Cleaved Caspase3 were examined. pAKT(Ser473) was not reliably detectable in Huh7 cells under the experimental conditions and was therefore not subjected to densitometric quantification. Calnexin was used as a loading control. Results are representative of three independent experiments and are expressed as the means ± SD (ns: not significant compared with the untreated control).

We investigated whether the optimized CuNP concentrations (0.04 µg/mL for Huh7 and 0.1 µg/mL for Mahlavu) induce programmed cell death. Acridine orange/ethidium bromide (AO/EB) dual staining was utilized to assess morphological changes associated with apoptosis. As shown in Figure 6A,C, cells treated with the respective optimized doses exhibited uniform green fluorescence and organized nuclear structures, comparable to the untreated control groups. Quantitative analysis confirmed that the percentage of apoptotic cells remained below 5% in both Huh7 and Mahlavu lines, with no significant difference observed between the control and treated groups (*p* > 0.05). To confirm these findings at the molecular level, we performed Western blot analysis to examine the expression of apoptotic markers and survival signaling proteins. Treatment with the optimized doses did not result in the induction of cleaved caspase-3, the primary executioner of apoptosis, in either cell line (Figure 6B,D). Furthermore, we assessed the status of the AKT signaling pathway, a key regulator of cell survival. In Huh7 cells, total AKT was detectable, whereas pAKT(Ser473) was not reliably detectable under the experimental conditions, precluding quantitative assessment of the pAKT(Ser473)/AKT ratio (Supplementary Figure S1). In Mahlavu cells, pAKT(Ser473)/AKT levels were not significantly altered following CuNP treatment. Collectively, these morphological and biochemical data demonstrate that at optimized concentrations, CuNPs do not trigger apoptotic cascades or compromise cellular homeostasis.

### 3.6. Optimized Non-Cytotoxic Concentrations of CuNPs Maintain Cellular Morphological Integrity in HCC Cell Lines

**Figure 7:**
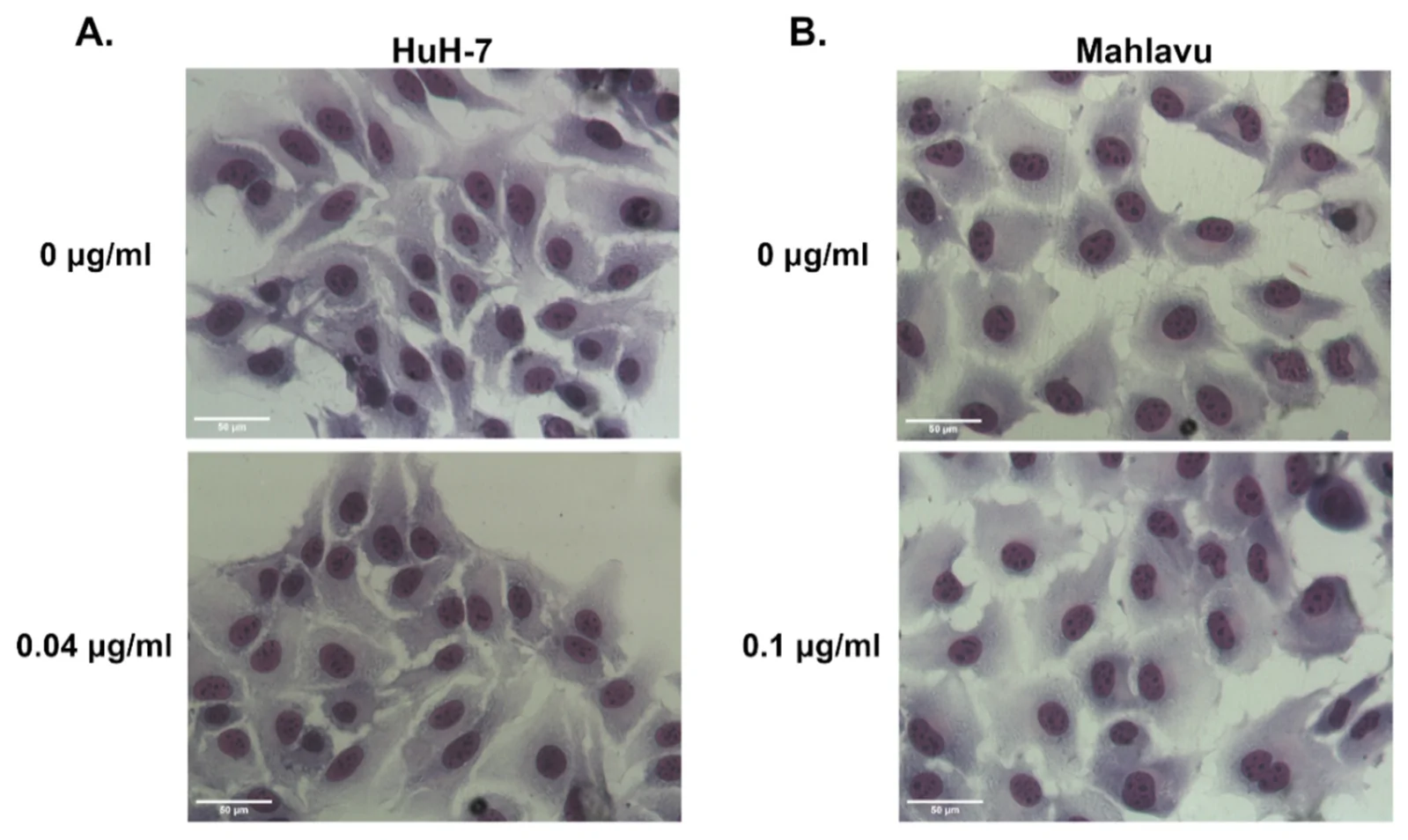
Optimized CuNP concentrations do not alter the morphological characteristics of Huh7 and Mahlavu cells. Representative light microscopy images of Giemsa-stained Huh7 (**A**) and Mahlavu (**B**) cells. Cells were treated with their respective optimized non-cytotoxic doses (0.04 µg/mL for Huh7 and 0.1 µg/mL for Mahlavu) for 120 h. The images demonstrate that the CuNP-treated cells maintain a morphology identical to the untreated control, with intact cellular boundaries and nuclear structures.

To further characterize the cellular effects of the selected concentrations at a microscopic level, we examined the morphological features of Huh7 and Mahlavu cells using Giemsa staining following treatment with optimized CuNP concentrations. Huh7 cells treated with 0.04 µg/mL CuNPs exhibited typical epithelial-like morphology, characterized by intact cell membranes and well-defined, prominent nuclei, showing no discernible differences compared to the untreated control group (Figure 7A). Similarly, the structural integrity of the poorly differentiated Mahlavu cell line remained visually uncompromised after exposure to 0.1 µg/mL CuNPs (Figure 7B). In both cell models, there was no evidence of cellular shrinkage, cytoplasmic vacuolization, or nuclear fragmentation, which are indicative of cytotoxicity or early apoptotic changes. Collectively, these morphological observations indicate that, at the selected concentrations, CuNPs exerted minimal detectable effects on cellular morphology.

## 4. Discussion

Nanotechnology has emerged as an important platform in modern medicine, enabling advances in drug delivery, molecular imaging, diagnostics, and targeted therapeutic strategies [27]. In oncology, nanoparticle-based drug delivery systems offer several potential advantages over conventional formulations, including improved solubility and stability, prolonged systemic circulation, enhanced biodistribution, and increased tumor-specific drug accumulation [28,29]. Among the various nanomaterials developed for biomedical applications, metallic nanoparticles have attracted particular interest because of their unique physicochemical properties and high surface area-to-volume ratios [30,31,32,33].

Copper is an essential trace element involved in cellular metabolism and energy production [34]. Notably, altered copper homeostasis and increased copper requirements have been reported in several malignancies, reflecting the metabolic demands of rapidly proliferating tumor cells [35]. These characteristics have stimulated interest in copper-based therapeutic approaches and copper-containing nanomaterials [36]. CuNPs, in particular, have been investigated both for their intrinsic biological activity and as potential drug-delivery platforms [16].

The physicochemical and biological properties of CuNPs are strongly influenced by their synthesis conditions. In the present study, CuNPs were synthesized by chemical reduction using NaBH_4_ as the reducing agent and PVP as the stabilizing agent. The morphology of the resulting nanoparticles was consistent with previous studies using similar synthesis approaches. Aguilar et al. reported that NaBH_4_-mediated reduction in the presence of PVP favored the formation of predominantly semi-spherical CuNPs and limited surface oxidation through stabilization of the nanoparticle surface [22]. Similarly, Khalid et al. obtained nanoscale copper particles of approximately 14–55 nm using NaBH_4_-mediated chemical reduction [37]. Guzman et al. further demonstrated that the concentration of NaBH_4_ and the presence of PVP influence nanoparticle nucleation, size distribution, and morphology [38].

The cytotoxicity of CuNPs varies considerably across experimental models, with reported effective concentrations ranging from the µg/mL to mg/mL range depending on particle size, synthesis conditions, surface characteristics, exposure duration, and the biological properties of the target cells [39,40,41]. Considerable variability has been observed even within the same tumor type. In HepG2 HCC cells, for example, reported IC50 values include 7 µg/mL, 19.88 µg/mL, and 13 µg/mL in studies by Song et al., Hassanien et al., and Li et al., respectively [20,21,42]. In the present study, the IC50 values were substantially lower, at 1.266 µg/mL in well-differentiated Huh7 cells and 1.498 µg/mL in poorly differentiated Mahlavu cells. These findings suggest greater cytotoxic potency of the CuNPs synthesized in our study compared with previously reported CuNP formulations in HCC models. This difference may reflect variations in nanoparticle size, morphology, surface characteristics, synthesis conditions, exposure parameters, and intrinsic biological differences among HCC cell lines.

On the other hand, the tissue- and cell-specific nature of CuNP toxicity is further illustrated by IC50 variations across different cancer models. For example, IC50 values of CuNPs have been reported as 68 µg/mL in SW480 human colorectal adenocarcinoma cells [43]; 73, 250, 142, and 115 µg/mL in NCI-H1975, NCI-H661, NCI-H1563, and NCI-H1573 human lung cancer cell lines, respectively [44]; 0.1 µg/mL in ovarian cancer cell lines and 0.14 µg/mL in MCF-7 breast cancer cells [45]. This broad range of IC50 values highlights that cytotoxicity is influenced not only by the physicochemical properties of the nanoparticles but also by cellular metabolic activity and intrinsic defense mechanisms. However, IC_50_ values across different cell lines and experimental studies are not directly equivalent and should be interpreted with caution. In this context, our data indicate that Mahlavu cells, an aggressive HCC model, exhibit a higher IC50 value than Huh7 cells, suggesting a more resistant phenotype against CuNP-induced cell death.

The vast majority of studies in the nanotechnology literature focus on the direct use of nanoparticles as anticancer agents and their cytotoxic mechanisms at high doses [9,12,46]. However, for nanoparticles to serve as effective drug or gene delivery vehicles, it is essential to define a safe dose range in which the nanoparticles themselves remain biologically inert, independent of the loaded therapeutic agent. This critical parameter has frequently been overlooked [47]. If a delivery vehicle impairs cellular homeostasis, triggers Caspase-3 activation, or suppresses cell proliferation on its own at therapeutic doses, it becomes impossible to distinguish whether the observed cytotoxic effect originates from the drug or the carrier. This ambiguity may complicate the interpretation of therapeutic efficacy and hinder the accurate assessment of nanoparticle-based drug delivery systems. Therefore, defining a biocompatible concentration range in which the carrier itself exerts minimal biological effects is essential for the reliable evaluation of nanoparticle-based drug delivery systems [48]. Our study directly addresses this gap by identifying CuNP concentrations of 0.04 µg/mL for Huh7 and 0.1 µg/mL for Mahlavu cells at which cell viability remained above 95% for up to 120 h, while colony-forming capacity and cellular morphology were preserved.

Although the MTT assay is widely used to evaluate in vitro nanoparticle cytotoxicity, it provides limited information on the effects of prolonged exposure [49]. Colony formation assays therefore provide a complementary approach for assessing long-term cellular survival and proliferative capacity [50,51]. In our study, exposure to the defined CuNP concentrations resulted in no significant reduction in colony formation compared with untreated controls. Consistent with these findings, AO/EtBr staining showed predominantly uniform green fluorescence with preserved nuclear morphology, and no significant increase in the proportion of apoptotic cells was observed.

At cytotoxic concentrations, particularly at or above IC50 levels, CuNPs have been reported to induce marked morphological alterations, including cell shrinkage, detachment, membrane blebbing, loss of epithelial morphology, cell aggregation, and chromatin condensation [52,53]. In contrast, HCC cells exposed to the concentrations evaluated in our study showed no detectable morphological alterations and maintained their overall structural integrity. Collectively, these findings indicate that, at the selected concentrations, CuNPs exert minimal effects on long-term clonogenic survival, apoptosis, and cellular morphology, supporting their potential suitability as drug-delivery platforms.

The PI3K/AKT/mTOR signaling pathway is a central intracellular pathway regulating cell survival, growth, differentiation, cellular metabolism, and cytoskeletal reorganization [54]. Dysregulation of the PI3K/AKT pathway during HCC progression confers survival and proliferation advantages to tumor cells while promoting resistance to apoptosis [55]. Previous studies have shown that cytotoxic concentrations of CuNPs can suppress PI3K/AKT signaling in various cancer cell models, leading to reduced cell survival, cell-cycle arrest, and increased caspase-3 activation [56]. Although these intrinsic effects may contribute to the anticancer activity of CuNPs, they may confound the interpretation of therapeutic responses when CuNPs are used as drug-delivery platforms, making it difficult to distinguish the effects of the loaded therapeutic agent from those of the carrier itself. Minimizing the intrinsic biological activity of the nanoparticle carrier is therefore important for accurately evaluating the activity and optimizing the dose of the delivered therapeutic agent. In our study, CuNPs at the selected concentrations did not significantly alter pAKT(Ser473)/AKT levels in Mahlavu cells or cleaved caspase-3 levels in Huh7 and Mahlavu cells under the experimental conditions. In contrast to the AKT inhibition and caspase activation reported at cytotoxic CuNP concentrations, these findings indicate that the selected CuNPs concentrations exert minimal effects on key survival and apoptotic signaling pathways in HCC cells. Together with the preserved cell viability, clonogenic capacity, and cellular morphology observed in our experiments, these molecular findings further support the potential suitability of CuNPs as drug-delivery platforms at the identified concentrations. The identified CuNP concentrations provide a defined starting point for future studies to determine the corresponding carrier-to-payload ratio. However, the actual drug-loading capacity of the CuNPs was not experimentally determined, and the low CuNP concentrations identified in this study may limit the amount of drug payload that can be delivered. Therefore, further studies are required to evaluate drug loading and release, cellular uptake, targeting properties, and the therapeutic efficacy of CuNPs loaded with appropriate anticancer agents.

Several limitations of the present study should be acknowledged. Although XRD, XPS and zeta-potential analyses could provide additional information on the crystalline phase, surface chemistry, oxidation state, and electrokinetic properties of the CuNPs, the primary objective of the present study was to investigate their biological effects in HCC cells. Accordingly, physicochemical characterization focused on particle morphology and size using SEM and DLS, whereas the biological responses were evaluated in detail using complementary cellular and molecular assays. Therefore, the scope of the present study was limited to the investigated biological endpoints and the morphological and size-related information obtained through SEM and DLS. Thus, no definitive conclusions could be drawn regarding crystalline phase, oxidation state, surface charge, PDI, or Cu ion release, as these parameters were not directly determined. Overall, the findings indicate that the NaBH_4_/PVP system produced CuNPs with morphological and size characteristics suitable for biological evaluation. Despite the evaluation of CuNPs in two different HCC cell lines in the present study, more comprehensive in vitro and in vivo toxicity analyses, including appropriate non-tumor cell models, are needed to better characterize the biological effects and safety profile of CuNPs. Such studies will be important for assessing their potential effects on normal cells and tissues, as well as systemic toxicity, and for determining their translational applicability. A further limitation is that pAKT(Ser473) was not reliably detectable in Huh7 cells under the experimental conditions, precluding quantitative assessment of pAKT(Ser473)/AKT signaling in this cell line. In addition, further studies using specific markers of apoptosis, cuproptosis, and other regulated cell death pathways are needed to more comprehensively characterize the mechanisms underlying the cellular effects of CuNPs. Matched DMSO vehicle controls were included at concentrations equivalent to those present in the corresponding CuNP-treated groups; however, the potential contribution of the vehicle to the observed biological responses should still be considered.

## 5. Conclusions

In conclusion, copper nanoparticles were successfully synthesized using a chemical reduction method and exhibited morphological and particle size characteristics suitable for subsequent biological evaluation. At the selected concentrations, CuNPs did not significantly affect cell viability, apoptosis, clonogenic capacity, cellular morphology, or key survival and apoptotic signaling pathways in HCC cell lines. These findings indicate that CuNPs can exhibit minimal intrinsic biological activity at appropriately selected concentrations, supporting their further investigation as potential drug-delivery platforms for HCC. Further studies evaluating CuNPs loaded with therapeutic agents, followed by in vivo assessment of systemic toxicity, biodistribution, and metabolic fate, are warranted to evaluate their effects and translational potential.

## Data Availability

The data presented in this study are available from the corresponding author on reasonable request.

## Supplementary Materials

The following supporting information can be downloaded at: https://www.mdpi.com/article/10.3390/biomedicines14102212/s1, Figure S1: Original Western blot images of pAKT(Ser473), total AKT in Huh7.

## Abbreviations

The following abbreviations are used in this manuscript:

CuNPs	Copper nanoparticles
NaBH_4_/PVP	Sodium borohydride/Polyvinylpyrrolidone
HCC	Hepatocellular carcinoma
SEM	Scanning electron microscopy
DLS	Dynamic light scattering
DMEM	Dulbecco’s Modified Eagle Medium
DMSO	Dimethyl sulfoxide
MTT	(3-(4,5-dimethylthiazol-2-yl)-2,5-diphenyl-2H-tetrazolium bromide)
IC50	The half-maximal inhibitory concentration
PBS	Phosphate-buffered saline
RIPA buffer	Radioimmunoprecipitation assay buffer
ECL	Enhanced chemiluminescence
