# Supplementary material for: Copper Nanoparticles at Optimized Concentrations Exhibit Minimal Intrinsic Biological Effects in Hepatocellular Carcinoma Cells
Source: Biomedicines. 2026 Sep 30;14(10):2212. doi: 10.3390/biomedicines14102212 (PMC13701396; doi:10.3390/biomedicines14102212)
Supplement: Supplementary file 1 [file biomedicines-14-02212-s001.zip › biomedicines-4553742-supplementary.pdf]

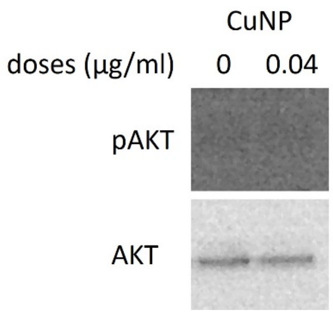

Figure S1. Original Western blot images of pAKT(Ser473), total AKT in Huh7
